# CLMP Is Essential for Intestinal Development, but Does Not Play a Key Role in Cellular Processes Involved in Intestinal Epithelial Development

**DOI:** 10.1371/journal.pone.0054649

**Published:** 2013-02-27

**Authors:** Christine S. van der Werf, Nai-Hua Hsiao, Siobhan Conroy, Joana Paredes, Ana S. Ribeiro, Yunia Sribudiani, Raquel Seruca, Robert M. W. Hofstra, Helga Westers, Sven C. D. van IJzendoorn

**Affiliations:** 1 Department of Genetics, University of Groningen, University Medical Centre Groningen, Groningen, The Netherlands; 2 Department of Cell Biology, University of Groningen, University Medical Centre Groningen, Groningen, The Netherlands; 3 The Cancer Genetics Group, Institute of Molecular Pathology and Immunology of the University of Porto, Porto, Portugal; 4 Department of Clinical Genetics, Erasmus University Rotterdam, Erasmus Medical Centre, Rotterdam, The Netherlands; University of Nevada School of Medicine, United States of America

## Abstract

Loss-of-function mutations in *CLMP* have been found in patients with Congenital Short Bowel Syndrome (CSBS), suggesting that its encoded protein plays a major role in intestinal development. CLMP is a membrane protein that co-localizes with tight junction proteins, but its function is largely unknown. We expressed wild-type (WT)-CLMP and a mutant-CLMP (associated with CSBS) in human intestinal epithelial T84 cells that, as we show here, do not produce endogenous CLMP. We investigated the effects of WT-CLMP and mutant-CLMP proteins on key cellular processes that are important for intestinal epithelial development, including migration, proliferation, viability and transepithelial resistance. Our data showed that expression of WT-CLMP or mutant-CLMP does not affect any of these processes. Moreover, our aggregation assays in CHO cells show that CLMP does not act as a strong adhesion molecule. Thus, our data suggest that, in the *in vitro* model systems we used, the key processes involved in intestinal epithelial development appear to be unaffected by WT-CLMP or mutant-CLMP. Further research is needed to determine the role of CLMP in the development of the intestine.

## Introduction

CLMP (coxsackie- and adenovirus receptor-like membrane protein) is a membrane protein that belongs to the CTX (cortical thymocyte marker in *Xenopus*) family of proteins [1]. The precise function of CLMP is largely unknown although several suggestions have been made. For instance, it has been suggested that CLMP plays a role in immunological processes [1], [2]. This is based on the fact that there is a high homology between CLMP and Junctional adhesion molecules (JAMs), both belonging to the CTX family of proteins. It is known that JAMs are important for transmigration of leukocytes to inflammatory sites [3]. This hypothesis was further supported by the finding that TNFα, a pro-inflammatory cytokine, is able to regulate *CLMP* expression [2]. In addition, it has been suggested that CLMP plays a role in cell-cell adhesion, based on the finding that it co-localizes with the tight junction proteins zonula occludens 1 (ZO-1) [1], [4], [5] and occluding [1]. Moreover, transfection of human *CLMP* in Chinese Hamster Ovary cells (CHO) induces cell aggregation [1], [6]. In addition, transfection of human *CLMP* into Madin-Darby canine kidney (MDCK) epithelial cells induces transepithelial electrical resistance (TER), suggesting a role for CLMP in the junction-barrier function of intestinal epithelial cells [1].

Loss-of-function mutations in *CLMP* were identified in patients with Congenital Short Bowel Syndrome (CSBS) [4]. A missense mutation was identified (V124D) in one of these CSBS patients. Transient transfection of this mutant-CLMP (*CLMP* containing the missense mutation V124D) in CHO and T84 cells resulted in mislocalization of CLMP and in an increased cytoplasmic pool of ZO-1 [4]. As tight junction proteins like ZO-1 play a role in cell proliferation [7], [8], it has been suggested that loss-of-function of CLMP would probably affect proliferation of human small intestinal cells during foetal development and thereby causing a shortened small intestine [4]. As the function of CLMP is still obscure, we aimed to gain a better understanding of the functional cellular role of CLMP.

## Materials and Methods

### Construction of plasmids for transient transfection of Chinese Hamster Ovary cells

A pCMV6-CLMP-green fluorescent protein (GFP) vector was obtained from Origene (Rockville, MD, USA). The *CLMP* missense mutation (c.730T>A, p.V124D) was introduced in this vector by site-directed mutagenesis (Stratagene, Amstelveen, Santa Clara, CA, USA) (for primer sequences see our previous publication) [4]. The wild-type (WT) and mutant cDNA were amplified using the primers CCGCC-*Nhe*I, 5′-ATGTCCCTCCTCCTTCTCC-3′, and GGGCGC-*Xho*I, 5′-TCAGACCGTTTGGAAGGCTCTG-3′. The amplification was performed using Phusion High-fidelity DNA polymerase (Finnzymes, Helsinki, Finland). The PCR products were inserted into PCR 2.1-TOPO plasmid (Invitrogen, Carlsbad, CA, USA). The PCR 2.1 Topo constructs were digested by *Nhe*I and *Xho*I restriction enzymes and the fragments were cloned into the vector pCMV-internal ribosomal re-entry site (IRES) coupled to eGFP (GFP-like protein). The clones were checked by direct sequencing.

### Construction of viral vectors for transduction of T84 cells

The pCMV-IRES-EGFP vectors, which were constructed as described above and contained both WT- and mutant cDNA (c.730T>A, p.V124D) of *CLMP*, were used as a template for amplification using the primers ACCA-*Nco*I-Myc, 5′- ATGTCCCTCCTCCTTCTC-3′ and AACA-*Xho*I- 5′-TCAGACCGTTTGGAAGGCTCTG-3′. The PCR products were digested with *Nco*I and *Xho*I and the fragments were cloned into the vector pEntr4. The clones were checked by digestion with *Nco*I and *Xho*I and direct sequencing. The inserts were subsequently cloned into the vector pLenti-CMV-Neo using lambda phage-based site-specific recombination and the Gateway® recombination cloning technology (Invitrogen).

### Production of lentiviral CLMP

For the production of the viruses, 2.6×10^6^ HEK293 cells were grown in Dulbecco's modified Eagle medium (DMEM) supplemented with 10% heat-inactivated foetal bovine serum (FBS, Invitrogen), 1% antibiotic solution (penicillin–streptomycin, Invitrogen) and 1% sodium pyruvate. The cells were maintained at 37°C in a humidified atmosphere with 5% CO_2_. Co-transfection of both WT and mutant pLenti-CMV-CLMP (V124D)-Neo with pVSV-G and pCMVdR8.1 was performed using the CaCl_2_ method. The cells were subsequently grown overnight. After 24 hours the medium was changed to DMEM supplemented with 10% heat-inactivated foetal bovine serum (FBS, Invitrogen) and 1% antibiotic solution (penicillin–streptomycin, Invitrogen). After 24 and 48 hours, the medium containing the virus was collected and stored at 4°C. Fresh medium was added to the cells. The collected medium was filtered using a polyvinylidene difluoride membrane-based filter to remove HEK293 cells.

### Cell culture

Chinese hamster ovary (CHO) and human intestinal epithelial T84 cells were grown in commercially available alpha modification of eagle's medium and DMEM/F-12 (both Invitrogen) respectively, supplemented with 4.5 mg/L L-glutamine, 10% heat-inactivated foetal bovine serum (FBS, Invitrogen) and 1% antibiotic solution (penicillin–streptomycin, Invitrogen). The cells were maintained at 37°C in a humidified atmosphere with 5% CO_2_.

### Production of stably transduced CLMP T84 cell lines

**Figure 1:**
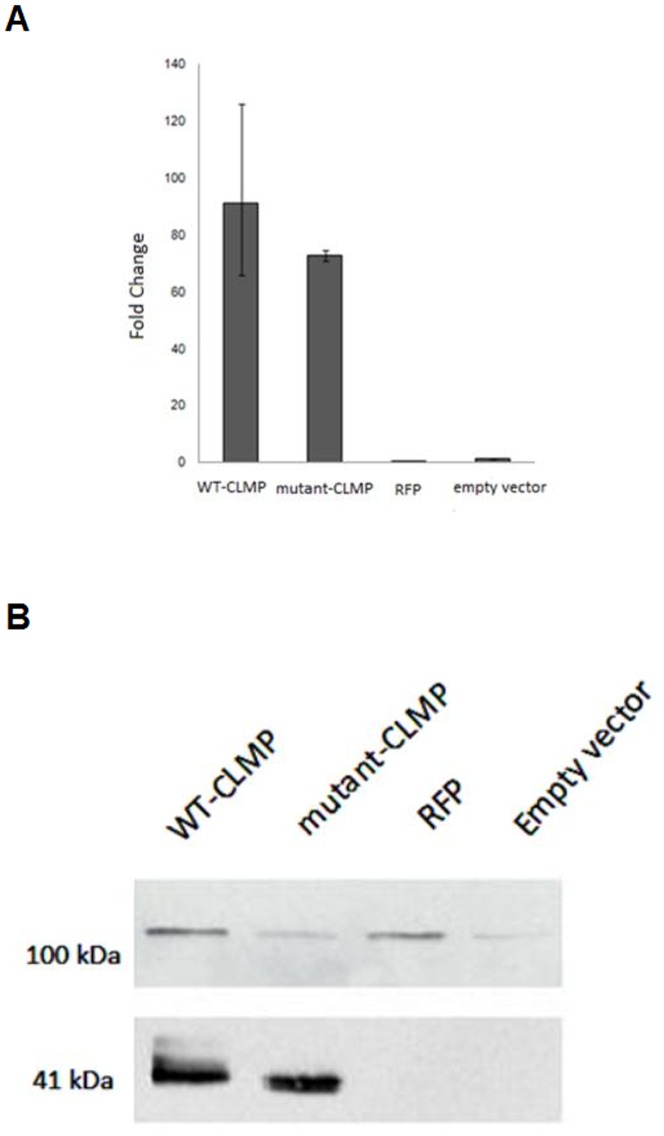
Expression of CLMP in T84 cells transduced with wild-type (WT)-CLMP, mutant-CLMP (V124D), RFP and an empty vector. There is no endogenous expression of CLMP in T84 cells (see the right lane (empty vector)). A. WT-CLMP and mutant-CLMP (V124D) mRNA were equally expressed in the transduced T84 cells as measured by real-time PCR. B. Western blots showed that WT-CLMP and mutant-CLMP (V124D) (at 41 kDa) protein were equally expressed. The 100 kDa band is an aspecific band derived from the vector.

For transduction the virus-containing solution and Hexadimethrine Bromide (Sigma; 4 mg/ml PBS) were added to T84 cells. The cells were then grown overnight at 37°C in a humidified atmosphere with 5% CO_2_. After 24 hours the medium was changed and the cells were grown till a confluency of 80% was reached. Transduction efficiency was checked by qPCR and Western blot analysis (see figure 1A).

### Real-time PCR

Expression of *CLMP* in the transduced T84 cells was quantified with Quantitative Polymerase Chain Reaction (qPCR). Transduced T84 cells were lysed and mRNA was isolated according to the manufacturer's instructions (GeneJET™ RNA Purification Kit, Fermentas). The *GAPDH* gene was used as an internal standard for normalization. mRNA was used as a template to synthesise cDNA. PCR was performed using the primers (CLMP-F) 5′-GAAGGAAAGCTGTGTGGTG- 3′ and (CLMP-R) 5′-CACTATGCCTGTCACTGCTC-3′ for *CLMP* and (GAPDH-F) 5′-CATTTCCTGGTATGACAACG- 3′ and (GAPDH-R) 5′-GTCCAGGGGTCTTACTCCTT- 3′ for *GAPDH* and the following amplification program: 15 minutes 95°C, 40 cycles 15 seconds 95°C, 1 minute 60°C. Each amplification reaction was run in triplicate using 10 ng of cDNA, 150 nM of both forward and reverse primers, and 1× SYBR green master mix (ABCM-221/A, Westburg, Leusden, the Netherlands) in a total volume of 10 µL. The results were analysed by StepOne^tm^ software v2.2 and recalculated manually using the Comparative C_T_ Method.

### Western blotting

Cells were harvested with lysis buffer (100 mM NaCl, 20 mM Tris-HCl pH 7.6, Triton X-100 and protease inhibitors (Roche, Almere, the Netherlands)). After incubation on ice for 30 minutes, the lysate was centrifuged for 5 minutes at 14,000 rpm at 4°C. Protein concentrations were determined using the BCA protein assay (Pierce Biotechnologies, Rockford, IL, USA) and measured on a NanoDrop® ND-1000 (Thermo Scientific, Waltham, MA, USA).

Protein extracts (40 µg) were resolved on an SDS/15% polyacrylamide gel, transferred on to a nitrocellulose membrane and blocked with dried milk powder in Tris-buffered saline with 0.1% Tween 20 for 1 hour at room temperature. The membrane was then incubated with primary antibody rabbit polyclonal antibody for CLMP (anti-AP000926.6, Sigma-Aldrich) in 1∶500 dilution for 1 hour at room temperature. After 1 hour incubation with the secondary antibody goat anti-rabbit conjugated with Horseradish peroxidase (1∶1,000; Bio-Rad, Hercules, CA, USA) at room temperature, the proteins were visualized using enhanced chemiluminescence reagent (Lumi-Light Western Blotting Substrate, Roche).

### Scratch/wound healing assay

Control T84 cells or T84 cells expressing WT-CLMP or mutant-CLMP (V124D) were cultured on glass-bottom petridishes (1.5×10^5^ per dish) for 7 days after which they develop a polarized monolayer with functional tight junctions. The dish was mounted on a microscope for live imaging. Monolayers were scratched with a micropipette and incubated in serum-deprived culture medium. After 24 hours of incubation, the rate of migration into the scratch was determined and presented as µm/24 hours. Experiments were performed in triplicate and data were expressed as mean ± SD.

### BrdU Cell Proliferation Assay

Cell proliferation was measured using a BrdU cell proliferation assay (Cell Signalling Technologies, Danvers, MA, USA) that detects 5-bromo-2′-deoxyuridine (BrdU) incorporated into cellular DNA during cell proliferation using an anti-BrdU antibody. T84 cells (1.5×10^5^) (control, WT-CMP or mutant-CLMP (V124D)) were plated and cultured for 2 days. BrdU was included in the culture medium at a final concentration of 10 µM and added to a monolayer of T84 cells (control, WT-CMP or mutant-CLMP (V124D)). After 24 hours, the labelling medium was removed, cells were fixed and BrdU incorporation was measured according to the manufacturer's instructions. Experiments were performed in triplicate and data were expressed as mean ± SD.

### XTT cell viability assay

Cell viability was measured using an XTT cell viability assay kit (Cell Signalling Technologies), a colorimetric assay that detects cellular metabolic activities that only occur in viable cells. T84 cells (1.5×10^5^) were plated and cultured for 2 days. The yellow tetrazolium salt XTT was then added to a monolayer of T84 cells (control, WT-CMP or mutant-CLMP (V124D)) at a final concentration of 20 µg/ml. After 4 h of incubation, the formazan dye that formed was quantified by measuring the optical density (OD) at wavelength 450 nm using a spectrophotometer. The OD measured at wavelength 690 nm was used as background reference. The specific OD was calculated by substracting the OD measured at wavelength 690 nm from the OD measured at wavelength 450 nm. Experiments were performed in triplicate and data were expressed as mean ± SD.

### Measurement of transepithelial electrical resistance in monolayer T84 cultures

T84 cells were grown on polycarbonate 24-wells Transwell filter inserts (Corning Costar, Corning, NY, USA) for 7 days. Transepithelial electrical resistance was measured using an epithelial Volt/Ohm-meter (World Precision Instruments, Sarasota, FL, USA). Experiments were performed in triplicate and data were expressed as mean ± SD.

### Transfection of CHO cells for aggregation assays

WT or mutant pCMV-CLMP (V124D)-IRES-EGFP was transfected into CHO cells (1.5×10^5^) with Lipofectamine 2000 Transfection Reagent (Invitrogen) in a 1∶3 dilution, and transfection efficiencies were evaluated by measuring EGFP expression by flow cytometry (around 50% efficiency).

### Aggregation assays

The bottoms of the wells of a 96-well plate were covered with semi-solid agar medium (100 mg Bacto-agar in 15 ml Ringer's salt solution) to prevent cell-substratum adhesion. Thereafter, a single cell suspension was added and incubated at 37°C in a humidified atmosphere with 5% CO_2_ for 24 hours and 48 hours. Aggregation was evaluated under an inverted microscope with a 4× objective and pictures were taken at both time points.

## Results

### Viral transduction of T84 cells

T84 colonic adenocarcinoma cells grow to confluent polarized monolayers that exhibit functional tight junctions and have been used extensively as a model to study intestinal tight junction integrity [9]–[11]. We found that T84 cells do not endogenously express CLMP (Figure 1), which makes this cell line a suitable model system to explore the effect of CLMP and CSBS-related CLMP mutants on epithelial functions related to the tight junction. For this, we stably transduced both WT-CLMP and mutant-CLMP (CLMP containing the missense mutation V124D). WT-CLMP and mutant-CLMP (V124D) were equally expressed in the transduced T84 cells as measured by real-time PCR (see Figure 1A). This result was confirmed by Western blot (see Figure 1B).

### CLMP does not affect migration of T84 cells

**Figure 2:**
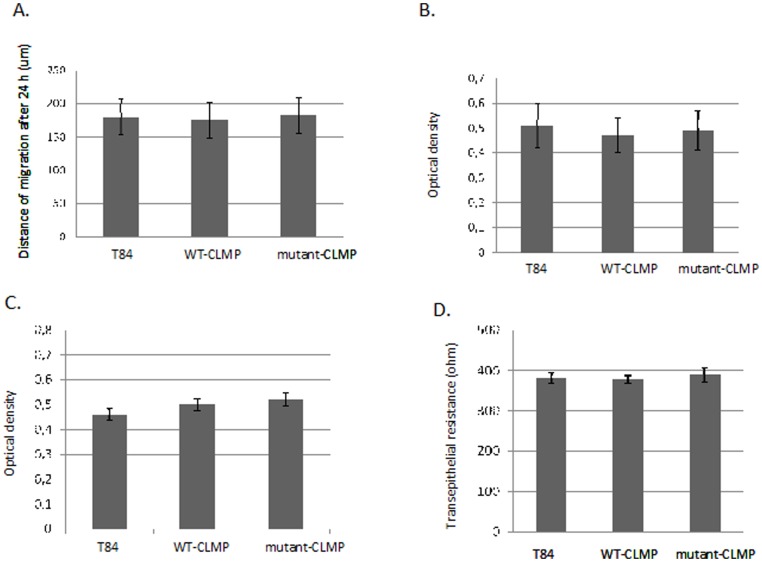
Overexpression of wild type (WT)-CLMP and mutant-CLMP (V124D) in human intestinal epithelial T84 cells does not affect wound healing/migration, cell proliferation, viability, and trans-epithelial electrical resistance. A. Cell monolayers were wounded and incubated in serum-deprived medium for 24 hours. The rate of directional cell migration (distance travelled/time unit) and wound closure were determined. B. Proliferation was quantified measuring BrdU incorporation using a BrdU cell proliferation assay. There was no significant difference in the specific optical density (OD) (the measured OD at wavelength 370 nm minus the measured OD at wavelength 492 nm) between the three groups. C. Cell viability was estimated using an *in vitro* Toxicology Assay Kit XTT-based. XTT was added to the cells at a final concentration of 20 µg/ml. After 4 hours of incubation the OD was measured at 450 nm using the 690 nm absorbance as the background. There was no significant difference between the three groups. D. Transepithelial electrical resistance was measured in confluent cell monolayers cultured on Transwell filter inserts.

To assess whether CLMP plays a role in intestinal cell migration, we performed a wound healing experiment. T84 cell monolayers were wounded and incubated in serum-deprived medium for 24 hours. There was no significant difference in the rate of directional cell migration (distance travelled/time unit) between the three groups (see Figure 2A). We concluded that overexpression of WT-CLMP or mutant-CLMP (V124D) does not affect the directed migration of T84 cells.

### CLMP does not interfere with proliferation of T84 cells

Proliferation of control and WT-CLMP or mutant-CLMP (V124D) expressing cells was quantified by measuring BrdU incorporation. There was no difference in BrdU incorporation between the three groups (see figure 2B). These data show that overexpression of WT-CLMP or mutant-CLMP (V124D) does not interfere with T84 cell proliferation.

### CLMP does not affect cell viability in T84 cells

To assess whether CLMP improved cell viability, we performed the *in vitro* XXT-based Toxicology Assay. T84 cells (1.5×10^5^) were plated and cultured for 2 days. After 4 hours of incubation with 20 µg/ml XTT, the specific absorbance was not significantly different between the three groups (see figure 2C). These data demonstrate that overexpression of WT-CLMP or mutant-CLMP (V124D) does not affect the viability of the T84 cells.

### CLMP does not increase transepithelial electrical resistance in T84 cells

Transepithelial electrical resistance is a measurement of ion flux over a polarized epithelial monolayer and can be used as a model for the junction-barrier function of tight junctions. Others have shown that overexpression of CLMP in MDCK cells, which do not express CLMP endogenously, increases the transepithelial resistance. We therefore assessed whether transfection of human CLMP into T84 cells influenced the transepithelial resistance. However, we observed no significant difference in transepithelial resistance between the control (not transduced) T84 cells, the T84 cells transduced with the WT-CLMP virus and the T84 cells transduced with the mutant-CLMP (V124D) virus (see Figure 2D). Thus, WT-CLMP and mutant-CLMP (V124D) do not increase or interfere with the transepithelial electrical resistance of a T84 cell monolayer.

### CLMP does not act as a strong cell-cell adhesion molecule

**Figure 3:**
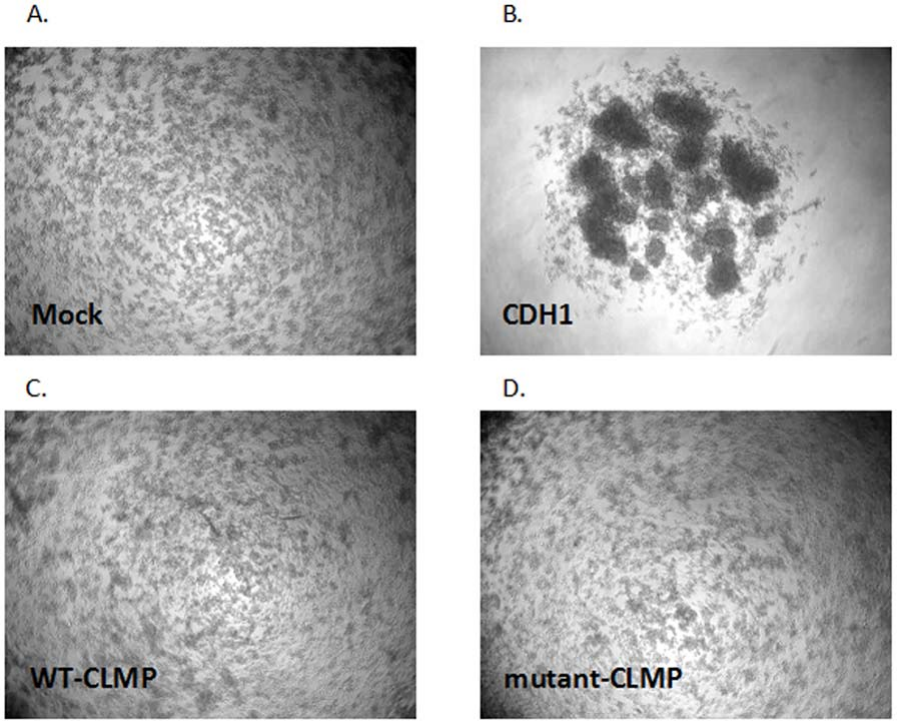
No significant difference was observed in cell aggregation between CHO cells transfected with and without CLMP (wild-type and mutant (V124D)). A. Mock, CHO cells that were not transfected. B. CHO cells transfected with CDH1. C. CHO cells transfected with wild-type-CLMP. D. CHO cells transfected with mutant-CLMP (V124D).

To determine the ability of CLMP to cause cell-cell adhesion, and the effect of the missense mutation (V124D) on its ability to do so, we decided to perform a slow aggregation assay, using CHO cells that do not aggregate at all in this assay. As a positive control, we transfected CHO cells with a *Cadherin 1* or also called epithelial or *E-cadherin (CDH1)* vector (which is a known cell-cell adhesion protein) which resulted in large aggregates (see Figure 3). However, there were no significant differences between non-transfected and transient human CLMP (wild-type or mutant (V124D)) transfected CHO cells after 24 hours or 48 hours of incubation (see Figure 3). We concluded that CLMP does not act like CDH1 as a strong cell-cell adhesion molecule.

## Discussion

Loss-of function mutations in *CLMP* were found in CSBS patients [4]. These patients have a congenital short small intestine with a mean length of 50 cm compared to a normal length of 250 cm at birth. CLMP is a trans-membrane protein and co-localizes with the tight junction proteins ZO-1 [1], [4], [5] and occluding [1]. Immunostaining on human embryos showed that CLMP was expressed in many tissues including the gut. Knock down experiments of the orthologue in zebrafish resulted in general developmental defects including an affected intestine. Goblet cells are normally present in the mid intestine in zebrafish (which resembles the small intestine in humans), and can therefore be used as a marker for this epithelial tissue. Since the goblet cells were absent in the morphant zebrafish, knock down of the orthologue of *CLMP* in zebrafish would probably result in the absence of the small intestine. All these findings suggest that CLMP has an important role in intestinal development, although its function is still largely unclear [4]. However, it is known that transient transfection of human CLMP into human intestinal epithelial T84 cells showed CLMP localization at the cell-cell membrane contacts [4]. It is also known that CLMP co-localizes with tight junction proteins, and is therefore claimed as a tight junction-associated protein. Because tight junction proteins play an important role in proliferation [7], [8], we have suggested that loss-of-function of CLMP might affect proliferation [4]. Moreover, it was shown that transfection of human *CLMP* into MDCK cells increases transepithelial resistance [1]. Whether it is proliferation, or transepithelial resistance, or indeed another process in which CLMP plays a crucial role and that has impact on the pathophysiology of CSBS, is still unknown.

To elucidate the function of CLMP we performed several functional assays using T84 cells as a model. As previously reported, transient transfection of human CLMP into T84 cells showed localization of WT-CLMP at the cell membrane and mislocalization of mutant-CLMP (V124D) in the cytoplasm [4]. Although others have shown that transfection of human CLMP into MDCK cells increases transepithelial electrical resistance [1], we did not observe any differences in the transepithelial electrical resistance in T84 cells. We cannot say whether this discrepancy is due to the use of distinct cell types (MDCK versus T84) or to the fact that CLMP is simply not involved in this process. MDCK cells are kidney cells derived from a seemingly normal adult female cocker spaniel. Many different strains of the MDCK cell line are available and the transepithelial resistance in these different strains differs depending on the tight junction proteins that are expressed [12]. This illustrates that even in the same cell line, different results can be obtained. Unfortunately, Raschperger *et al* did not mention which strain of the MDCK cell line they used [1]. T84 cells are human colon carcinoma cells that have been widely used to study intestinal epithelial function [9]–[11]. We would therefore like to argue that T84 cells form a more representative model for studying the function of CLMP than MDCK cells.

We reported earlier that the missense mutation in *CLMP*, which was identified in one of the CSBS patients, leads to CLMP cytoplasmatic mislocalization [4]. To study whether this missense mutation would also affect the adhesion properties of CLMP, we performed a cell aggregation assay using a widely accepted protocol implemented by Boterberg *et al* (Metastasis Research Protocols) [13]. As a positive control we transfected CHO cells with cadherin 1, also called epithelial or E-cadherin, a cell-cell adhesion protein that is an important component of the adherens junction [14], [15]. In our assay, transfection of CDH1 resulted in large aggregates, but we did not observe wild-type CLMP acting as a strong cell-cell adhesion protein. In the previous reports that claim that CLMP acts as an adhesion protein, the assays were performed differently. Raschperger *et al* used CAR as a positive control [1]. Eguchi *et al* used low- and high-*CLMP* expressing stably transfected cell lines and observed a significant difference in the size of the aggregates [6]. The incubation time that Raschperger *et al* and Eguchi *et al* used in the aggregation assays was shorter, 60 minutes and 90 minutes compared to the incubation time of 24 hours and 48 hours we used in our experiments. Differences in the formation of aggregations might be found in a shorter time frame, while they probably become equal after a longer incubation time. This might well explain why we were not able to confirm the previous findings. However, when we compare our figures with those of both Raschperger *et al* and Eguchi *et al*, we notice that the size of the aggregates we observed in CHO cells transfected with CDH1 was far bigger. In addition, the figures in the previous reports are much closer to our figures for the CLMP-transfected CHO cells than to our positive control, CHO cells transfected with CDH1. CLMP might have some adhesion capacity comparable with CAR, but based on our assays we have to conclude that CLMP is not a strong adhesion molecule like CDH1.

Because CLMP co-localizes with tight junction proteins and tight junction proteins play an important role in proliferation [7], [8], we thought that loss-of-function of CLMP might affect proliferation [4]. However, in our proliferation assay in T84 cells, we could not elucidate a role for CLMP in proliferation and based on our assays in T84 cells, CLMP also does not play a role in migration and cell viability.

From our results we have to conclude that CLMP does not play a major role in cell-cell adhesion, nor does it affect migration, proliferation, cell viability and transepithelial electrical resistance in T84 cells. The fact that T84 cells do not express CLMP, but do form proper tight junctions, points to the conclusion that CLMP cannot be essential for tight junction formation. Thus, although CLMP co-localizes with tight junction proteins, its function is probably not directly related to the tight junction. More research is needed to better understand the function of CLMP and why loss-of-function mutations in *CLMP* cause Congenital Short Bowel Syndrome.
